# Artificial Ex Utero Systems to Treat Severe Periviable Fetal Growth Restriction—A Possible Future Indication?

**DOI:** 10.3390/jcm13226789

**Published:** 2024-11-11

**Authors:** Oluwateniayo O. Okpaise, Aaron J. Fils, Gabriele Tonni, Rodrigo Ruano

**Affiliations:** 1 Medway Maritime Hospital, Gillingham ME7 5NY, UK; teniayookpaise@gmail.com; 2 Miller School of Medicine, University of Miami, Miami, FL 33136, USA; ajf179@med.miami.edu; 3 Department of Obstetrics & Neonatology, Istituto di Ricovero e Cura a Carattere Scientifico (IRCCS), ASL di Reggio Emilia, 42122 Reggio Emilia, Italy; gabriele.tonni@ausl.re.it; 4 Division of Maternal-Fetal Medicine, Department of Obstetrics, Gynecology & Reproductive Sciences, Miller School of Medicine, University of Miami, Miami, FL 33136, USA

**Keywords:** fetal growth retardation, artificial organs, intensive care, neonatal, artificial placenta–uterus system

## Abstract

Fetal growth restriction, or intrauterine growth restriction, is a common gestational condition characterized by reduced intrauterine growth. However, severe periviable fetal growth restriction is still associated with elevated perinatal mortality and morbidity. The current literature advises delivery once it is deemed that fetal compromise is evident. As uteroplacental insufficiency is the most common etiology of this condition, we hypothesize that the use of artificial ex utero systems to provide adequate nutrition and recreate the uterine environment may be a viable treatment option in this situation, even with the possibility of treating severe fetal growth restriction and prevent sequelae. There are promising experimental studies in sheep models investigating the artificial ex utero system for potential prenatal conditions, but future additional investigation is needed before translating to clinical trials in humans.

## 1. Introduction

Fetal growth restriction (FGR), also known as intrauterine restriction (IUGR), is a condition that affects 5–10% of pregnancies globally and is a leading cause of perinatal mortality [1]. Various definitions of FGR exist; the World Health Organization defines it as fetal weight below the third percentile, while the American College of Obstetrics and Gynecology defines it as losing 10% of fetal weight at a given gestational age [1,2]. The etiology of FGR is multifactorial, with fetal, maternal, placental, and external factors contributing to impaired fetal growth and development [3]. While these factors contribute to this disease’s pathogenesis, placental dysfunction remains the major causative factor [3].

Periviability, the state of fetal development in which extrauterine survival is minimal, is determined as the timeframe between the 22nd and 26th week of gestation or those that weigh below 1000 g at birth [4,5]. High mortality rates mar this period, as these infants are suspectable to significant morbidities, including intraventricular hemorrhages, necrotizing enterocolitis, and bronchopulmonary dysplasia. These conditions can cause long-lasting sequelae and, in many cases, death [4].

As there are limitations to the perinatal management of severe periviable FGR with still elevated perinatal mortality, we hypothesize that artificial uterine–placental systems can be a future option in this situation.

## 2. Fetal Growth Restriction

### 2.1. Classifications

According to the Delphi Consensus ultrasound-based criteria, fetal growth restriction can be classified as early- or late-onset [6]. These criteria were determined following an international collaboration of FGR experts using a 5-point scale to assess the importance of parameters associated with fetal growth restriction [6].

Early-onset FGR is less prevalent, with an incidence rate of 30%; it is diagnosed before the 32nd gestational week and must include one of the following criteria for a confirmed diagnosis:

- Late changes to umbilical artery Doppler studies;
- Fetal abdominal circumference (AC) and estimated fetal weight (EFW) < 3rd percentile;
- Fetal AC or EFW < 10th percentile accompanied by abnormal uterine and umbilical Doppler studies [6,7].

Late onset accounts for the remaining 70% and is diagnosed after the 32nd gestational week. This diagnosis can be made if the abdominal circumference is < 3rd percentile alone or is associated with at least two of the following criteria:

- Fetal AC and EFW < 10th percentile;
- Umbilical artery pulsatility index (UAPI) > 95th percentile or a cerebroplacental ratio below the 5th percentile;
- An EFW or AC that crosses two quartiles [6,7].

While other societies, including the International Society of Ultrasound in Obstetrics and Gynecology (ISUOG) and the Society for Maternal-Fetal Medicine (SMFM), have accepted the Delphi criteria, these criteria do not take into account Doppler ultrasonographic findings but instead define FGR as EFW or AC below the 10th percentile [8]. Diagnostic tools, including cardiotocography (CTG) and short-term variation (STV), aid in the determination of the complications associated with FGR, including fetal hypoxia and alteration in heart rate, which may inform of impending fetal compromise [9].

While these subtypes can develop from similar pathological conditions, there are strong correlations between FGR subtypes and certain disease states. Early-onset fetal growth restriction has a strong correlation with placental dysfunction and fetal hypoxic state; this leads to fetal acidosis in early stages of the fetal life, which can cause severe injury and intrauterine death [10]. On the other hand, late-onset fetal growth restriction may be less severe since the postnatal management of these babies has better outcomes, despite the fact that late-onset fetal growth has a high risk of rapid deterioration, such that the fetus has an increased risk of intrauterine distress, stillbirth, and acidosis in the postpartum period [10].

### 2.2. Pathogenesis

Fetal growth restriction is the inability of a fetus to achieve its determined genetic growth potential. While factors like maternal nutrition, fetal genetic anomalies, and underlying infections can play a role in disease development, a strong correlation between placentation and FGR exists, and this is particularly present in early-onset fetal growth restriction [11].

Placental deficiency in FGR is thought to be associated with aberrant molecular signaling pathways that lead to a reduction in placental volume and vasculature [11]. The main receptors found to be associated with fetal growth restriction include hypoxia-induced factor (HIF), vascular endothelial growth factor (VEGF), placental growth factor (PIGF), and tyrosine kinase receptor 1 (Flt-1) [12]. These receptors are responsible for gene expression related to angiogenesis, or the formation and maintenance of placental vasculature [12]. The aberrant expression or downregulation of the receptors named above prompts vascular inadequacy, causing inadequate placental gas exchange and oxygenation, which prompts fetal hypoxia and, eventually, growth restriction [12].

Cellular proliferation in the placenta is controlled by insulin-like growth factor (ILGF), which, through a cascade, activates the AKT/mTOR pathway and allows for the translation of mRNA into protein, prompting placental growth through acellular proliferation and the expression of transporters needed for the transfer of essential amino acids, glucose, and fatty acids [11]. In fetal growth restriction, this pathway is disrupted through a combination of transporter downregulation and hypoxia.

The hypoxic state is hypothesized to be secondary to maternal vascular insufficiency due to aberrant spiral artery remodeling caused by inadequate interstitial trophoblast invasion and placental bed apoptosis [11]. As such, these vessels are highly prone to sclerotic changes, causing luminal narrowing and impairing the velocity of maternal blood flow as it enters the placental villous space [11].

### 2.3. Etiology

Chromosomal abnormalities account for approximately 7% of all cases of fetal growth restriction. Chromosomal aneuploidy, particularly trisomies 13, 18, and 21, will present with asymmetrical growth restriction as they typically have abnormal uterine artery indices [13]. Infections in utero, whether viral or parasitic in nature, impair fetal cell proliferation, causing an overall decline in fetal growth [13].

A positive correlation between growth impairments and fetal prematurity has long been determined [13]. This relationship is likely due to prematurity’s heterogeneity, as it shares common risk factors with growth restriction, including high parity, ethnic origins, etc. [14].

Multiparity is associated with a lag in growth curves, typically around the 30th to 32nd gestational week, compared to primiparous cohorts; lags occur earlier with higher fetal numbers [13].

Hypertensive disorders complicate up to 40% of FGR pregnancies, as vasculopathy causes diminished fetal nutrition and thus causes growth deficiencies [13]. These hypertensive conditions encompass disorders including pre-eclampsia, chronic hypertension, and autoimmune conditions causing endothelial dysfunction [13].

Maternal lifestyle choices, including the use of illicit drugs and cigarette smoking, can cause both fetal and maternal vasoconstriction due to circulating waste products like carbon monoxide, which interfere with fetal oxygenation, leading to decreased fetal weight and growth [13].

### 2.4. Periviability and FGR

Periviability is defined as birth between the 20th and 25th gestational weeks and has historically proved to be a high-risk situation leading to adverse fetal outcomes, including mortality and morbidity, as these infants have an extremely low birth weight (typically less than 1000 g) [15]. Uteroplacental insufficiency accounts for the development of periviability in approximately 90% of cases [16]. The combination of placental dysfunction and impaired fetal growth showed a perinatal mortality risk five to six times greater compared to infants of average growth, particularly for infants in the periviable period [17]. This increased mortality is in response to the hypoxia secondary to placental insufficiency. Using sheep models, Baschat [18] determined that when oxygen delivery falls below 0.6 mmol/min/kg of fetal body weight, this significantly reduces the fetal uptake of essential nutrients, including glucose, lipids, and essential amino acids, causing fetal malnutrition and insufficient fetal development. Impaired oxidative metabolism will cause lactate to accumulate, causing metabolic acidosis, which, in association with the underlying hypoxia, will additionally impair fetal circulation [18].

The combination of FGR and periviability leads to significant neonatal mortality and morbidities affecting multiple organ systems, especially when the estimated fetal weight is less than 500 g and in the case of absent/reverse end diastolic flow in the umbilical artery and absent/reverse A waive in the Ductus venosus [6,7,8,9,10].

The gastrointestinal tract is particularly susceptible as ongoing hypoxia causes the redistribution of blood flow away from the developing gut, leading to necrotizing enterocolitis (NEC) [19]. This hypoxia also leads to fetal oxidative stress and inflammation, which impairs hemodynamic development, leading to placental vascular resistance; in response, maladaptive cardiac development occurs, making these infants prone to cardiovascular disease later on in life [19]. Chronic hypoxia has been correlated with aberrant pulmonary development. Bronchopulmonary dysplasia (BPD), arrested airway, and parenchymal development have been linked to FGR 45% of the time [19]. Pulmonary hypertension can develop due to aberrant vascular development secondary to cardiac remodeling, impairing lifelong respiratory capacity [19]. FGR and periviability correlate with suboptimal neurological development, leading to cognitive, behavioral, and motor deficits. MRIs of fetal brains showed reduced brain volume and abnormal morphology due to distorted cortical folding stemming from demyelination and neuroinflammation secondary to hypoxia [19]. Due to severely low birth weight and fetal size, an increased risk of cerebral palsy is seen; intraventricular hemorrhage can also occur, presenting as hypotonia, seizures, or coma [19,20].

## 3. Artificial Placenta–Uterus Systems—Current Status

Artificial placentas, which in the past were a theoretical possibility for treating preterm fetuses, were first implemented by Westin et al. in 1958 [19]. They determined that periviable fetuses have decreased chances of survival when they are kept at 37 °C as there is increased tissue metabolism with limited nutrition sources [21]. They determined that administering oxygenated blood through the umbilical vein helped to stabilize these periviable fetuses and thus hypothesized that a continuous infusion of oxygenated blood could help to sustain these infants ex utero. With their perfusion technique, fetuses were sustained for up to 12 h [21].

Since then, different artificial placenta models have been developed in an attempt to prolong fetal survival ex utero and encourage continued fetal development and growth.

During the development of early models, the challenge of providing adequate nutrition arose. Early models only provided sugars; modern systems deliver 70–80 kcal/kg/day by providing amino acids, lipids, vitamins, and sugars [22]. However, a balance must be maintained because continual caloric-rich feed can propagate hyperglycemia and have diabetic-like effects on the fetus, while reduction can exacerbate inadequate growth [22].

In order to replicate adequate utero-placental physiology, a pumpless model is ideal, as the pump used by the Westin required significant pressure to overcome high cardiac afterloads secondary to the flow resistance of the oxygenator, which led to fetal cardiac decompensation, causing fetal hydrops and circulatory compromise [22]. To compensate, newer models include open-top reservoirs to counteract the pulsatile flow of blood [17]. In addition, early systems used film oxygenators, but direct blood–air contact increased the risk of infection. Modern systems use hollow-fiber oxygenators, which have resistance equivalent to placental resistance, thus removing the need for pumps and the direct exposure of blood to air [22]. Artificial placenta–uterus models submerge the fetus in a warm, sterile fluid environment (~39 °C) [22]. This fluid replicates amniotic fluid by insulating the fetus from sound and mechanical pressure, maintaining body temperature, protecting the umbilical vessels, and preventing insensible fluid loss; it stimulates lung development and allows for fetal breathing and swallowing of fluid [22]. The previously used open and semi-closed systems allowed for bacterial proliferation and were plagued by infections; modern systems utilize closed environments with fluid exchange, significantly reducing infection rates [22].

Since the era of Westin, success has been achieved in sustaining fetuses in artificial placental and womb models. There is a current artificial placental model, known as the artificial placenta, was developed at the University of Michigan. Their model utilized endotracheal fetal (ET) intubation, rather than the typical fluid immersion, to support fetal breathing and lung development [22]. The artificial placenta used a pump circuit to maintain physiological fetal circulation, and, as stated above, significant cardiac decompensation and even cardiac arrest were common causes of fetal compromise [22]. The fetal lambs used were 118–130 days old and weighed approximately 2.5 to 5.1 kg, which is an age equivalent to human fetuses in the periviable period; survival on the circuit was noted for up to 17 days [22]. Additional details about all systems can be found in Table 1. Due to this, future studies should strive to move towards pumpless circuits in order to bypass these complications.

The artificial uteri in development are the ex vivo uterine environment (EVE) and the extra-uterine environment for neonatal development (EXTEND). The EVE was developed under joint collaboration with the Sendai team from Japan and Australia; they aimed to target the systemic and organic dysfunction seen in earlier studies, which they did by introducing hydrocortisone to their protocol. The EVE supported fetal lambs at 112–115 days’ gestation for up to seven days. To determine the efficacy of this model in sustaining periviable fetuses, another trial with the EVE was conducted with fetal lambs at 95 gestational days, weighing 0.6–0.7 kg, in the hopes of stimulating human fetuses at the 22–24th week age range; survival, in this case, was only five days and there was evidence of white matter injury.

The EXTEND, developed in Philadelphia, supported lamb fetuses aged 106–117 days on the circuit for up to 28 days [22]. Their model used a pumpless arterio-venous system and involved submerging the lambs in sterile fluid with continuous exchange of synthetic amniotic fluid. During their study, they found that they did not find the need to employ the use of corticosteroids to aid in lung maturation [22]. Not only did lung development match that of age-related cohorts, but continual fetal development was noted, with the cardiac and neurological systems showing ongoing progression. Notably, some lambs survived for up to 6 months once weaned off the circuit [23]. Recent tests in lambs at 90–95 days gestation weighing 0.69–1.31 kg sustained lambs for up to 21 days [23]. Before beginning the study, three lambs were incidentally found to have FGR at 105–108 days’ gestation, weighing approximately 0.82kg, and were sustained for up to 23 days [23]. At autopsy, they weighed 1.18–1.76 kg, demonstrating continued growth, helping to establish the plausibility that growth-restricted fetuses can be sustained on an artificial placenta–uterus system.

## 4. Conclusions

As these systems have highlighted, artificial placental models have great potential to support growth-restricted fetuses, particularly in the periviavle stage with an estimated fetal weight of less than 500 g and absent end diastolic flow in the umbilical artery and absent/reverse A waive in the Ductus venosus, as the primary causative factor is uteroplacental insufficiency [24]. These systems can potentially avoid perinatal demise and FGR as a whole, as continued fetal growth has been deemed apparent in animal trials. In addition to providing nutrition, these systems offer environmental conditions to support continued organ development and maturation, unlike current therapies. As oxygenated blood is supplied to these fetuses, the oxidative stress associated with FGR-induced hypoxia is prevented, preventing the long-term sequelae of cardiovascular dysfunction later in life. Organic dysfunction is also preventable, as ongoing lung and neurological development have been proven in studies [23]. While still in the early stages of testing, the plausibility of these artificial systems being able to correct the morbidities associated with FGR could prevent the need for surgical and multidisciplinary intervention. Further animal studies are needed before this technology is converted from a theoretical setting to a clinical one in treating human fetuses.

## Figures and Tables

**Table 1:** Ex utero system characteristics [20].

	Artificial Placenta	EVE	EXTEND
Cannulation	Umbilical vein—jugular vein	Umbilical arteries—umbilical vein	Umbilical arteries—umbilical vein
Oxygenation	1 oxygenator with pump in parallel	2 oxygenators (no pump) in parallel	1 oxygenator (no pump)
Fluid submersion	No, ET tube filled with perfluorocarbons	Yes, exchanged every 6 h	Yes, continuous exchange
Complications leading to mortality	Cannula-related Cardiac arrhythmia and arrest Pericardial tamponade	Equipment failure Thromboembolism Cannula-related	Equipment failure, cannula-related Umbilical spasm Circuit clotting
Nutrition	TPN, dextrose	TPN, glucose, lipids (70–75 kcal/kg)	TPN, dextrose, lipids (80 kcal/kg)
Medications	Antibiotics Vasopressors Anticoagulation Corticosteroids PGE1 Erythropoietin Diazepam (PRN) Buprenorphine (PRN)	Antibiotics Anticoagulation Lipo-PGE1 Erythropoietin Milrinone (24 h)	Anticoagulation Erythropoietin PGE1 Insulin Buprenorphine (PRN) Propofol (PRN)

Abbreviations: EVE = ex vivo uterine environment, EXTEND = extra-uterine environment for neonatal development, ET = endotracheal intubation, TPN = total parenteral nutrition, PGE1= prostaglandin E 1, Lipo-PGE1 = liposomal prostaglandin E1, PRN = “pro re nata” meaning medications as needed.

## Data Availability

No new data were created or analyzed in this study. Data sharing is not applicable to this article.
